# *Staphylococcus aureus* lipid factors modulate melanoma cell clustering and invasion

**DOI:** 10.1242/dmm.050770

**Published:** 2024-09-16

**Authors:** Morgan A. Giese, Gayathri Ramakrishnan, Laura H. Steenberge, Jerome X. Dovan, John-Demian Sauer, Anna Huttenlocher

**Affiliations:** ^1^ Department of Medical Microbiology and Immunology, University of Wisconsin-Madison, School of Medicine and Public Health, Madison, WI 53706, USA; ^2^ Cellular and Molecular Biology Graduate Program, University of Wisconsin-Madison, Madison, WI 53706, USA; ^3^ Cancer Biology Graduate Program, University of Wisconsin-Madison, Madison, WI 53706, USA; ^4^ Department of Biochemistry, University of Wisconsin-Madison, Madison, WI, USA; ^5^ University of Wisconsin School of Medicine and Public Health, Madison, Wisconsin, USA; ^6^ Morgridge Institute for Research, Madison, Wisconsin, USA; ^7^ University of Wisconsin Medical Scientist Training Program (MSTP) Summer Scholars, University of Wisconsin-Madison, Madison, WI 53706, USA; ^8^ Department of Pediatrics, University of Wisconsin-Madison, Madison, WI 53706, USA

**Keywords:** Melanoma, Zebrafish, *S. aureus*, Lipids, Skin microbiome

## Abstract

The microbiome can influence cancer development and progression. However, less is known about the role of the skin microbiota in melanoma. Here, we took advantage of a zebrafish melanoma model to probe the effects of *Staphylococcus aureus* on melanoma invasion. We found that *S. aureus* produces factors that enhance melanoma invasion and dissemination in zebrafish larvae. We used a published *in vitro* 3D cluster formation assay that correlates increased clustering with tumor invasion. *S. aureus* supernatant increased clustering of melanoma cells and was abrogated by a Rho-Kinase inhibitor, implicating a role for Rho-GTPases. The melanoma clustering response was specific to *S. aureus* but not to other staphylococcal species, including *S. epidermidis*. Our findings suggest that *S. aureus* promotes melanoma clustering and invasion via lipids generated by the lipase Sal2 (officially known as GehB). Taken together, these findings suggest that specific bacterial products mediate melanoma invasive migration in zebrafish.

## Teaser

**Summary:** *S. aureus* supernatant increases invasion of melanoma cells in zebrafish and is likely to be mediated by the production of bacterial lipids.

## INTRODUCTION

The host microbiome is capable of influencing all aspects of health, including cancer initiation and progression (Zheng et al., 2020; Sun et al., 2023). Oncogenic bacteria (oncomicrobes), i.e. microbes with carcinogenic properties, cause an estimated 2.2 million cancer cases per year (IARC Working Group on the Evaluation of Carcinogenic Risks to Humans, 2012). Furthermore, there is a growing list of ‘complicit’ microbes that are capable of promoting cancer progression (Sepich-Poore et al., 2021). These microbes can alter proliferative versus cell-death signals, produce DNA-damaging toxins or induce cancer cell invasion by triggering epithelial-to-mesenchymal transition (EMT) (Garrett, 2015; Casasanta et al., 2020; Parhi et al., 2020). Additionally, microbes are highly capable of modulating the immune response, either promoting an inflammatory tumor microenvironment or an immunosuppressive environment that prevents tumor cell killing (Sepich-Poore et al., 2021; Garrett, 2015; Crusz and Balkwill, 2015). As the gut is the largest reservoir of microbes in the human body, most studies have focused on the impact of gut microbes on cancer development and therapy response (Iida et al., 2013; Gopalakrishnan et al., 2018). The skin represents the second largest microbiota population in the body, yet few studies have evaluated the role that these microbes play in cancer development.

Increasing evidence indicates that skin microbes, such as *Staphylococcus aureus,* are linked to cancers including cutaneous T-cell lymphoma (Yu et al., 2015; Squarzanti et al., 2020; Woo et al., 2022). *S. aureus* is commonly found on healthy skin and colonizes 20-40% of the general population (Swaney and Kalan, 2021), yet its presence is a leading risk for surgical site infections as *S. aureus* can transition to a pathogenic state with changes in the environment (Seidelman et al., 2023). Non-melanoma tumors were found to have an overabundance of *S. aureus* compared to healthy skin (Kullander et al., 2009; Wood et al., 2018), and challenge with *S. aureus* increased proliferation of squamous cell carcinoma (SCC) in humans (Madhusudhan et al., 2020). For melanoma, only one publication has profiled the microbial community on patients and found that *Propionibacterium*, *Staphylococcus* and *Corynebacterium* were the most common genera on both healthy skin and melanocytic lesions (Salava et al., 2016). In pig skin models, *Staphylococcus* species were more prevalent on cutaneous melanoma compared to healthy skin (Mekadim et al., 2022), but the effect of these bacteria on the melanoma microenvironment is unknown. With 100,000 predicted diagnoses in 2023, melanoma occurrence has steadily increased in the United States and accounts for the majority of skin cancer-related deaths (*American Cancer Society*). Given the correlation between *S. aureus* and the development of other skin cancers, we sought to determine the effect of *S. aureus* on cutaneous melanoma growth and progression.

Here we used a larval zebrafish model to investigate the impact of *S. aureus* on melanoma progression. Zebrafish models for cancer cell transplantation are well developed and allow for study of the early stages of cancer invasion (Weiss et al., 2022; Patton et al., 2021; White et al., 2008; Astell and Sieger, 2020). In this study, we showed that *S. aureus* produces factors capable of enhancing melanoma cell invasion and dissemination. To determine the mechanism driving melanoma invasion, we took advantage of an *in vitro* 3D cluster formation assay using ultra-low attachment plates. More invasive melanoma cells have previously been shown to form large spherical clusters due to increased expression of adhesion genes, correlating with increased metastasis in zebrafish (Campbell et al., 2021). We found that incubation with *S. aureus* supernatant increased clustering of melanoma cells, which was abrogated by inhibition of Rho-associated protein kinases 1 and 2 (ROCK1 and ROCK2, respectively). The clustering response was specific to *S. aureus* and not to other staphylococcal species, including *S. epidermidis*, or other Gram-negative bacteria tested. Furthermore, we determined that *S. aureus* may promote cluster formation via lipids generated by the lipase GehB (also known as and hereafter referred to as Sal2), as genetic mutation resulted in reduced melanoma clustering and invasion. Therefore, these findings suggest that *S. aureus* produces lipids that promote melanoma invasion.

## RESULTS

### *S. aureus* supernatant promotes melanoma invasion and dissemination ***in vivo***

**Fig. 1.**
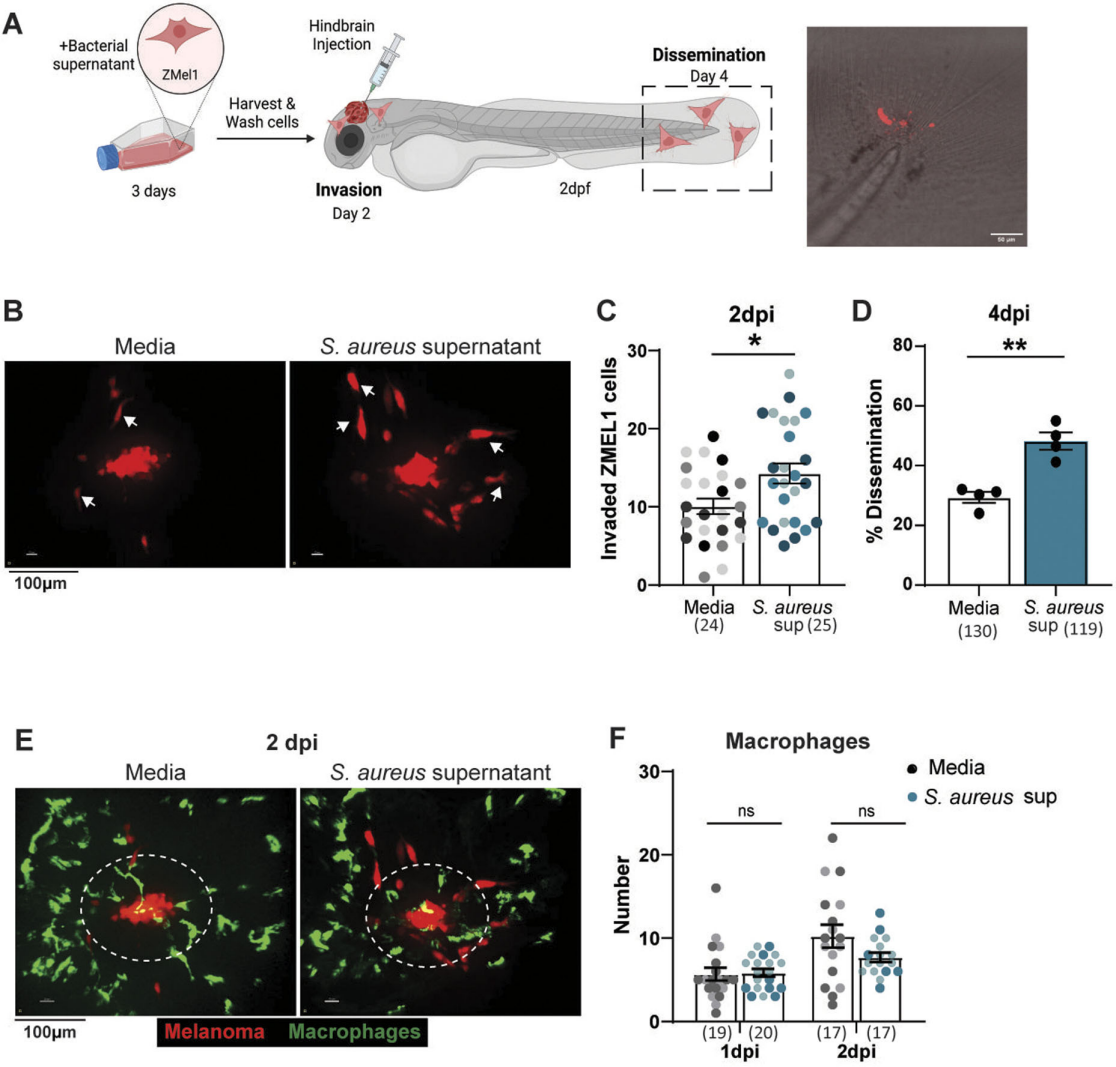
***S. aureus* supernatant promotes melanoma invasion and dissemination *in vivo*.** (A) Schematic of ZMEL1-td-Tomato melanoma cell injection into larval zebrafish hindbrain, created with BioRender.com. Representative image of ZMEL1 dissemination to tail fin of 4dpi zebrafish larvae. Confocal imaging was performed in the hindbrain at 2 days post injection (dpi) and in the tail at 4 dpi. (B,C) Representative images of ZMEL1 melanoma cell invasion (B) and quantification of ZMEL1 melanoma cell invasion (C) at 2 dpi in the zebrafish hindbrain after culture in medium alone (Media) or in *S. aureus* USA300 supernatant. Arrows in B indicate migrated melanoma cells with an elongated morphology. Results are from three independent experiments (*n*=24-25 larvae per condition). Dots in C represent independent larvae color coded per replicate. (D) Percent of zebrafish with disseminated ZMEL1 melanoma cells in the tailfin at 4 dpi. Results are from three independent experiments (*n*=130 larvae for medium control, *n*=119 larvae with *S. aureus* supernatant). Dots represent independent replicates. Error bars indicate the mean±s.e.m. (E,F) Representative images of macrophage recruitment to ZMEL1 melanoma cells (E) and quantification of macrophage recruitment to ZMEL1 melanoma cells (F) at 2 dpi. Areas surrounded by dashed line indicate the 50-μm region within which recruited immune cells were counted Results are from two independent experiments (*n*=17-20 larvae per condition). Error bars indicate the mean±s.e.m.. *P*-values were calculated using unpaired *t*-test (C), paired *t*-test (D) or two-way ANOVA (F). *P<0.05; **P<0.01, ns, not significant.

To evaluate the effect of *S. aureus* on melanoma cell behavior *in vivo*, ZMEL1-GFP zebrafish melanoma cells were incubated with *S. aureus* bacterial supernatant for 3 days, washed and then injected into the larval zebrafish hindbrain (Fig. 1A). Bacterial supernatants were used over live bacteria to limit bacterial overgrowth. The larval hindbrain had previously been used as a model to assess melanoma and other cancer cell dissemination *in vivo* (Weiss et al., 2022; Astell and Sieger, 2020). Larval zebrafish are optically transparent, which allows for *in vivo* visualization of cell behavior, including local cancer cell invasion and dissemination (Schoen et al., 2023; Roh-Johnson et al., 2017). Using fluorescent confocal imaging, we visualized melanoma invasion over time. Two days post injection (2 dpi), melanoma invasion away from the cell mass was increased after pre-incubation with bacterial supernatant, compared to culture in medium alone (Fig. 1B,C). Furthermore, ZMEL1 melanoma cells displayed an elongated morphology indicative of the mesenchymal morphology of single invading tumor cells (Fig. 1B) (Barros-Becker et al., 2017; Ribatti et al., 2020). Following migration into the tissue, cancer cells can invade into the skin or enter the vasculature to spread to other regions of the zebrafish (Campbell et al., 2021). We imaged cancer cell dissemination in the tail fin and found that melanoma cells cultured in *S. aureus* supernatant were significantly more likely to disseminate (Fig. 1D). Our findings show that pre-treatment of melanoma cells with *S. aureus* supernatant modifies melanoma cell behavior and increases invasion and dissemination into tissues.

We next wanted to determine if increased melanoma cell invasion is associated with increased immune cell infiltration. Increased recruitment of immune cells can promote cancer cell progression and metastasis (Gonzalez et al., 2018; Giese et al., 2019). Larval zebrafish have an intact innate immune system that can be live imaged to evaluate infiltration of immune cells and association with tumor cells (Roh-Johnson et al., 2017; Korte et al., 2022). Using fluorescent reporters for neutrophils and macrophages, we imaged innate immune cell recruitment to the injection site. Neutrophils were recruited early, arriving within hours of melanoma injection, but showed little interest in ZMEL1 melanoma cells. We found no difference in the number of neutrophils with *S. aureus* supernatant incubation versus medium alone (Fig. S1A, B). Macrophages are recruited secondarily and in greater number, with peak recruitment at 2 dpi. Similar to neutrophils, we found no significant difference in the level of macrophage recruitment to melanoma cells pre-treated with *S. aureus* supernatant (Fig. 1E,F). Therefore, ZMEL1 melanoma cell invasion and dissemination to the fin is likely to be due to direct effects of the supernatants on melanoma cells.

### *S. aureus* supernatant promotes melanoma clustering *in vitro*

**Fig. 2.**
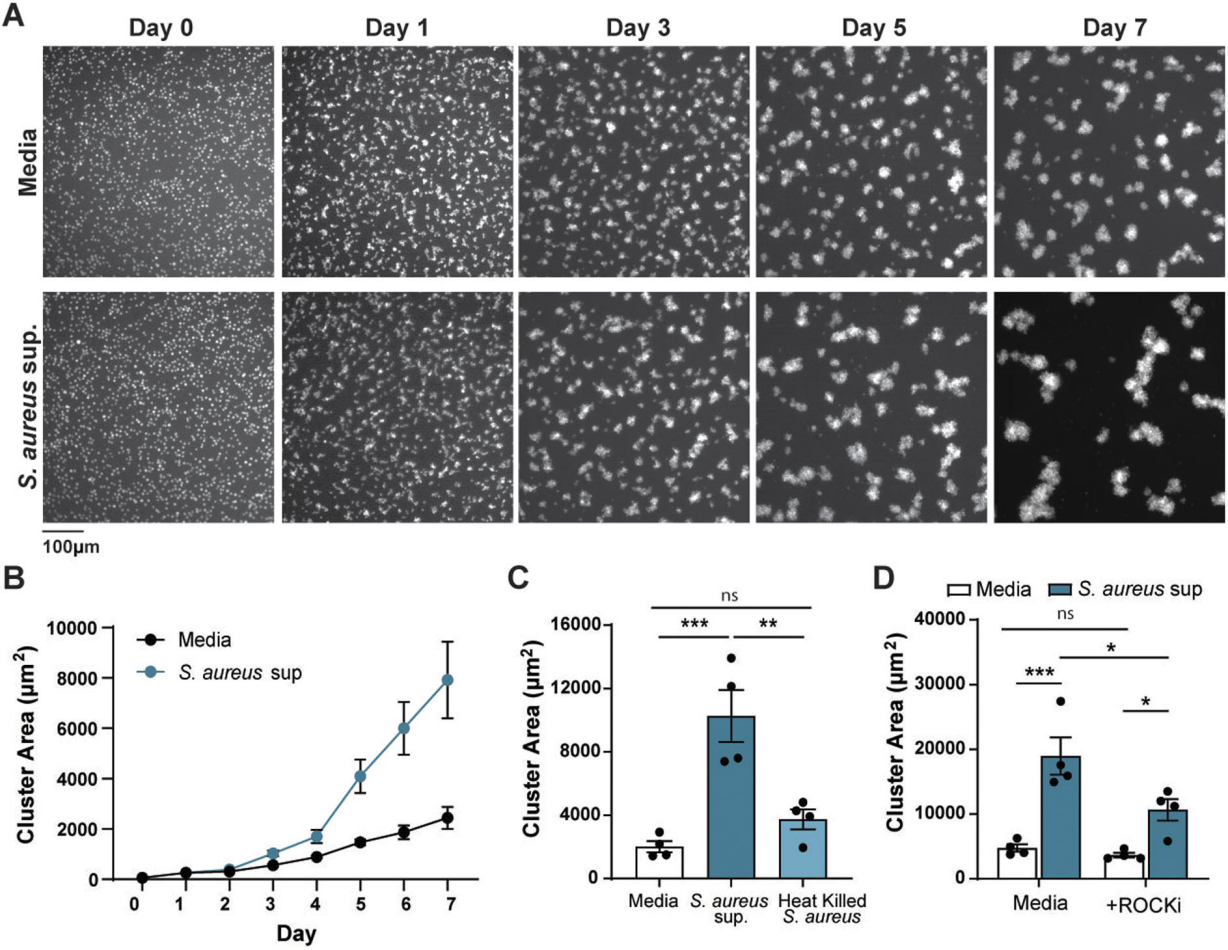
***S. aureus* supernatant promotes melanoma clustering *in vitro***. (A) Representative images of ZMEL1-EGFP melanoma cells cultures in the presence of medium alone (Media) or *S. aureus* USA300 supernatant imaged over 7 days. (B) Plot shows one representative experiment with quantification of melanoma cluster area (µm^2^) over time. Error bars indicate represent mean±s.e.m. (C,D) Melanoma cluster size (µm^2^) with *S. aureus* USA300 supernatant or heat killed bacteria (C) at 7 days or with *S. aureus* USA300 supernatant plus 500 nM of the ROCK inhibitor Y-27632 (D) at 7 days. Experiments were conducted at least three times. Dots in C and D represent independent replicates. Error bars indicate the mean±s.e.m. *P*-values were calculated by one-way ANOVA (C) or two-way ANOVA (D). *P<0.05; **P<0.01; ***P<0.001. ns, not significant.

To determine how *S. aureus* supernatant promotes melanoma invasion, we used a previously developed 3D *in vitro* clustering assay on ultra-low attachment plates (Campbell et al., 2021). Increased invasion and metastasis of ZMEL1 melanoma cells in zebrafish has been shown to correlate with enhanced *in vitro* clustering response due to increased cell-cell adhesion (Campbell et al., 2021). We tested the effect of *S. aureus* supernatant on melanoma cell clustering by imaging cells over the course of 7 days and found little difference in cluster size for the first 3 days of culture. However, after day 4, melanoma cell clusters exponentially increased in size when cultured in the presence of *S. aureus* bacterial supernatant (Fig. 2A, B). At day 7, *S. aureus* supernatant significantly increased melanoma cluster size compared to culture in medium alone (Fig. 2C). We further checked the viability of melanoma cells to determine if *S. aureus* supernatant provides a survival benefit over RPMI medium alone. For both conditions tested, we did not observe a significant difference in cell death during the culture period (Fig. S2A,B). To evaluate if *S. aureus* bacteria promote clustering, we co-cultured melanoma cells with equivalent colony-forming units (CFUs) of heat-killed *S. aureus*. We found a small increase in clustering compared with medium alone, but this was significantly reduced compared to supernatant (Fig. 2C). In summary, *S. aureus* supernatant is capable of producing secreted factors that promote melanoma clusters *in vitro*, correlating with the increased invasion observed *in vivo*.

Next, we live-imaged melanoma cells over time to determine if increased cluster size is, indeed, due to enhanced cell-cell adhesion, as previously described (Campbell et al., 2021). Melanoma cells cultured in bacterial supernatant were more active than cells in medium alone, and clusters were faster to migrate towards one another, promoting larger cluster formation ( Fig. S3A,B) (Movies 1 and 2). To determine if *S. aureus* supernatant promotes cluster formation due to increased migration of melanoma cells, we utilized the ROCK1/2 inhibitor Y27632 (hereafter referred to as ROCK inhibitor). ROCK1/2 act downstream of Rho GTPase to promote cytoskeletal rearrangement and cell motility (Amano et al., 2010). Furthermore, Rho signaling can promote cancer cell migration and invasion (Jeong et al., 2012). Addition of the ROCK inhibitor to melanoma cells cultured together with bacterial supernatant significantly diminished cluster formation (Fig. 2D). There was no effect of ROCK inhibitor on melanoma cells incubated with medium only (control), indicating that the ability to cluster was not affected. Therefore, our findings suggest that *S. aureus* supernatant promotes melanoma cell migration via activation of Rho-GTPase signaling.

### Melanoma clustering is specific to *S. aureus* species

**Fig. 3.**
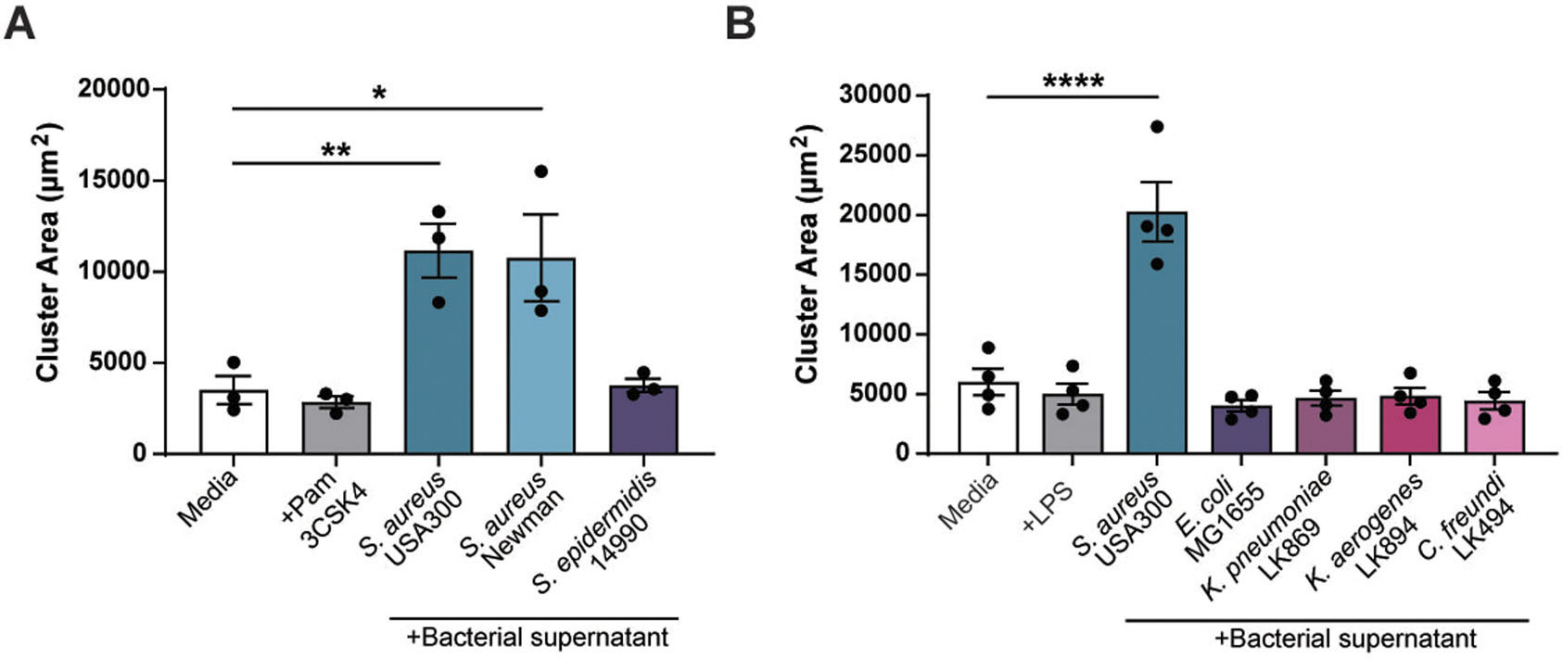
**Melanoma clustering is specific to *S. aureus* species.** Quantification of ZMEL1 melanoma cell cluster size (µm^2^) after 7 days of culture in either RPMI medium alone (Media) or in RMPI medium plus bacterial supernatant. (A) RPMI medium alone (Media), or with addition of Pam3CSK4 (100 µg/ml) or with one of the bacterial supernatants obtained from a selection of cultured Gram-positive bacteria as indicated was added to melanoma cell culture. (B) RPMI medium alone (Media), Media plus LPS (1 µg/ml) or with one of the bacterial supernatants obtained from a selection of cultured Gram-negative bacteria as indicated was added to melanoma cell culture. Experiments were conducted at least three times. Dots represent independent replicates. Error bars indicate the mean±s.e.m. *P*-values were calculated by one-way ANOVA. *P<0.05; **P<0.01; ****P<0.0001.

We next determined if melanoma clustering is specific to the methicillin-resistant *S*. *aureus* (MRSA) strain USA300 or whether it can be induced by a broader spectrum of bacteria. Gram-positive bacteria, such as *S. aureus*, are recognized by melanoma cells through the toll-like receptor TLR2 (Duan et al., 2022; Burns and Yusuf, 2014). We added the TLR2 agonist Pam3CSK4 but found no effect on melanoma cluster size, indicating that TLR2 activation alone is not sufficient to promote cluster formation (Fig. 3A). Next, we next tested *S. aureus* strain Newman, a methicillin-sensitive *S. aureus* (MSSA) strain with high genetic similarity to USA300 (Baba et al., 2008). *S*. *aureus* Newman was capable of promoting melanoma cluster formation at levels similar to those observed for *S*. *aureus* USA300 (Fig. 3A). The commensal species *Staphylococcus epidermidis*, however, did not induce clusters (Fig. 3A), suggesting that not all *Staphylococcus* species are capable of promoting melanoma clustering.

We also tested a selection of Gram-negative bacterium species, including three clinical isolates obtained from human skin (i.e. *K. pneumoniae*, *K. aerogenes*, *C. freundi*). None of the tested Gram-negative bacteria were capable of inducing melanoma cluster formation (Fig. 3B). Furthermore, addition of the TLR4 agonist LPS did not affect melanoma clustering. Taken together, these findings indicate that the effect of bacterial supernatant on melanoma clustering is specific to the *S. aureus* species tested and is not induced by TLR activation alone.

### *S. aureus* effect on melanoma clustering is likely to be mediated by lipids

**Fig. 4.**
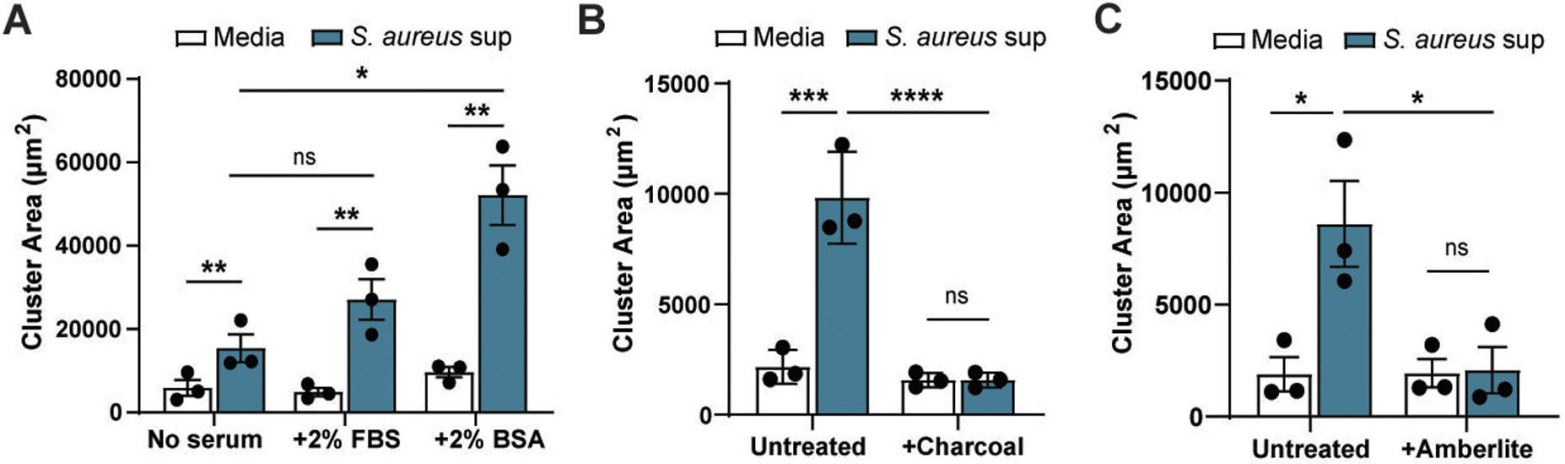
***S. aureus* effect on melanoma clustering is likely to be mediated by lipids.** Quantification of ZMEL1 melanoma cell cluster size (µm^2^) after 7 days of culture in medium alone or plus *S. aureus* USA300 bacterial supernatant. (A) Supernatants were collected from *S. aureus* USA300 bacteria grown in RPMI medium alone Media), or RPMI medium supplemented with either 2% FBS or 2% BSA followed by culturing with melanoma cells. (B) Supernatants collected from *S. aureus* USA300 bacteria grown in RPMI medium alone (Media) were left untreated or treated with dextran-coated charcoal (+Charcoal) followed by culturing with melanoma cells. (C) Supernatants collected from *S. aureus* USA300 bacteria grown in RPMI medium alone (Media) were left untreated or treated with Amberlite-XAD4 (+Amberlite) followed by culturing with melanoma cells. Experiments were conducted at least three times. Dots represent independent replicates. Error bars indicate the mean±s.e.m. *P*-values were calculated by two-way ANOVA. **P<0.01; ***P<0.001; ****P<0.0001. ns, not significant.

Next, we modified the culture medium used to grow *S. aureus* to determine the effect of supplemented medium components on melanoma clustering. Microbiota utilize available host components during infection, including *S. aureus* which incorporates human serum lipids into its membrane during pathogenesis (Hines et al., 2020). Thus, addition of host nutrients may influence production of the clustering factor by *S. aureus*. We generated supernatants from *S. aureus* grown in RPMI alone or supplemented with fetal bovine serum (FBS) or bovine serum albumin (BSA). While there was a trend towards larger clusters with the addition of FBS over serum-free medium, this was not significant (Fig. 4A). These data suggest that serum components do not affect *S. aureus* production of the clustering factor. Interestingly, addition of BSA to bacterial supernatant significantly enlarged melanoma cluster size, with almost a three-fold increase (Fig. 4A). BSA is often included as a medium supplement in serum-free medium as it promotes cell growth and survival. It does so by binding essential medium components including fatty acids and other lipids to increase their concentration and interaction and uptake into cells (van der Vusse, 2009; Francis, 2010). The increased clustering with BSA indicates that albumin may be able to bind hydrophobic molecules produced by *S. aureus* to enhance melanoma clustering. Furthermore, albumin proteins are present in FBS and may account for the slight increase in cluster size we observed with FBS supplementation.

To test if hydrophobic molecules in *S. aureus* supernatant are responsible for melanoma clustering, we stripped off the bacterial supernatant with dextran-coated charcoal, which completely abrogated melanoma cell clustering (Fig. 4B). Use of a second stripping method by using Amberlite XAD4 beads, which are highly adsorbent for hydrophobic compounds, also resulted in loss of the clustering phenotype (Fig. 4C). Thus, a hydrophobic molecule in *S. aureus* supernatant is likely to mediate clustering.

### Deletion of lipases in *S. aureus* alters melanoma clustering

**Fig. 5.**
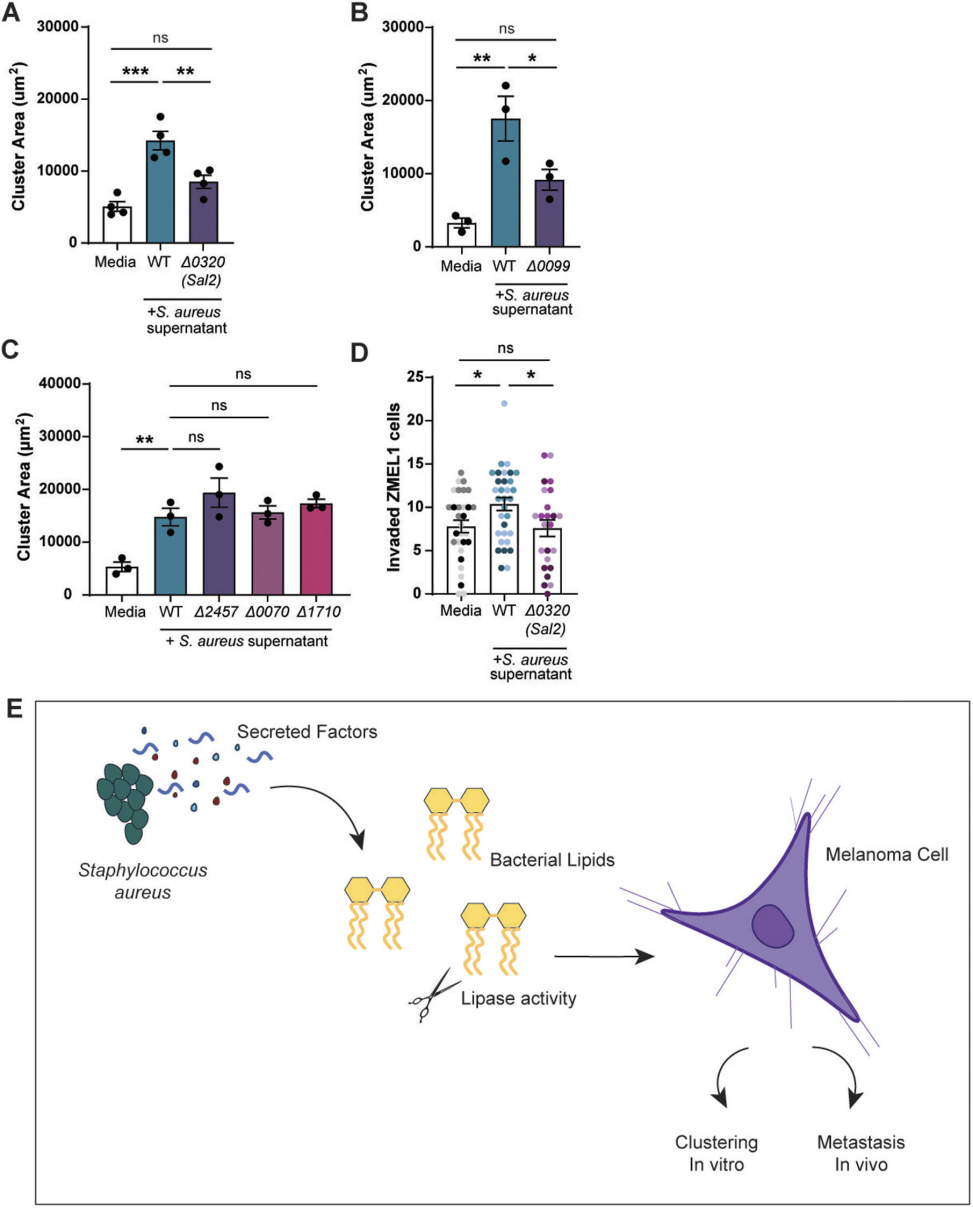
**Deletion of *S. aureus* lipases alters melanoma clustering and invasion.** Quantification of ZMEL1 melanoma cell cluster size (µm^2^) after 7 days of culture in RPMI medium alone or plus *S. aureus* USA300 bacterial supernatant as indicated. (A–C) Supernatants were tested from USA300 transposon mutants for lipases *Sal2*/*Geh* (Δ0320) (A), phosphatidylinositol-specific phospholipase C (Δ*0099*) (B) or for putative phospholipase genes Δ2457, Δ0070 and Δ1710 (C). PRMI medium alone (Media) was used as control. (D) ZMEL1 invasion at 2 dpi in the zebrafish hindbrain after culture in medium alone or in *S. aureus* USA300 supernatant. Dots represent independent larvae color coded per replicate. Medium control (*n*=30 larve); WT bacterial supernatant (*n*=32 larvae); Δ0320 supernatant (*n*=24 larvae). Experiments were conducted at least three times. Dots in A-C represent independent replicates. Error bars indicate the mean±s.e.m. *P*-values were calculated by one-way ANOVA (A–D). *P<0.05; **P<0.01; ***P<0.001. ns, not significant. (E) Schematic summarizing the findings described in this article, showing that *S. aureus* secreted factors promote melanoma clustering *in vitro* and invasion *in vivo*.

Bacteria synthesize hydrophobic molecules, particularly lipids, necessary for key cell functions, including formation of the cellular envelope (Hines et al., 2020; Ryan et al., 2023). Production of these lipids is mediated by a cascade of enzymes (Sohlenkamp and Geiger, 2016). Taking advantage of an available *S. aureus* USA300 transposon mutant library, we tested the ability of lipase mutants to promote melanoma clustering. A variety of lipases, including Sal2 (also known as GehB; SAUSA300_0320) are secreted by *S. aureus*. Sal2 has ester hydrolase activity, and cleaves both short and long chain triglycerides (Cadieux et al., 2014). We found that supernatant from *S. aureus* ΔSal2 significantly decreased cluster size (Fig. 5A), indicating that Sal2 activity produces lipids that promote clustering. Sequence alignment to *S. aureus* found no Sal2 ortholog in *E. coli*, *K. pneumoniae* or *K. aerogenes* (Altschul et al., 1990), which correlates with our clustering data. While *S. epidermidis* contains a Sal2 ortholog (Rosenstein and Gotz, 2000), its level of secretion is unclear.

To determine if the clustering effect is specific to Sal2, we also tested a selection of phospholipase mutants. Phosphatidylinositol-specific phospholipase C (PIPLC) (SAUSA300_0099) is produced and secreted by all *S. aureus* strains, but most highly expressed by the USA300 and Newman strains (White et al., 2014; Volwerk et al., 1990). Supernatant from the PIPLC mutant Δ0099 revealed a significant decrease in clustering compared to that of WT (Fig. 5B). Other putative phospholipase mutants (SAUSA300_2457, SAUSA300_0070, SAUSA300_1710) showed no significant effect on melanoma cluster size (Fig. 5C). These findings suggest that bacterial lipid products mediate melanoma clustering.

Finally, we wanted to test the effect of these lipase mutants on melanoma behavior *in vivo*. We pre-incubated ZMEL1 melanoma cells with ΔSal2 (Δ0320) *S. aureus* supernatant, injected the cells into the hindbrain of larval zebrafish and imaged melanoma invasion at 2 dpi. We found that melanoma cells incubated with this bacterial supernatant showed a significant decrease in invasion compared to those incubated with WT supernatant (Fig. 5D). Thus, *S. aureus* lipases are likely to produce lipids that induce melanoma cell clustering *in vitro* and invasion *in vivo*. Taken together, these findings suggest that *S. aureus* generates factors that are modified by bacterial lipases and that these products influence melanoma cell invasion (Fig. 5E).

## DISCUSSION

Changes in gut microbiota can affect cancer development, disease progression and response to therapy (Hanahan, 2022). The impact of the skin microbiota on carcinogenesis and cancer progression is less clear but increasing evidence indicates that these microorganisms are also capable of influencing the balance between skin health and disease (Savoia et al., 2023). In this study, we found that *S. aureus* supernatant promotes melanoma invasion and dissemination in a transplant larval zebrafish model. Furthermore, *in vitro* analysis determined that lipids produced by *S. aureus* are likely to promote melanoma migration through the activation of Rho-GTPases.

Bacteria can promote cancer progression by impacting immune cell function in the tumor microenvironment (Casasanta et al., 2020). Specifically, increased immune infiltration results in chronic or high-grade inflammation, and induces tumor cell invasion and metastasis (Garrett, 2015; Smith and Trinchieri, 2018). We found that zebrafish melanoma cells cultured with *S. aureus* bacterial supernatant were more migratory and invaded into the tissue *in vivo*. It is possible that some of the cluster size and invasion phenotypes were also mediated by changes in cell proliferation. However, we did not find a significant difference in cell numbers between conditions after *in vitro* culture (data not shown). We quantified innate immune cell recruitment to determine if there was an increased inflammatory response to ZMEL1 cancer cells that had been pre-treated with *S. aureus* bacterial supernatant, but found no difference in neutrophil or macrophage numbers. Alternatively, *Fusobacterium nucleatum* promotes migration of  colorectal cancer cells by increasing secretion of inflammatory chemokines directly from the cancer cells themselves (Casasanta et al., 2020). Melanoma cells express TLRs and initiate NF kappa B signaling, including production of chemotactic factors (Burns and Yusuf, 2014; Sharma et al., 2010; Wang et al., 1990). Thus, *S. aureus* might induce autocrine signaling of inflammatory chemokines to promote tumor cell migration.

Cancer metastasis is often associated with epithelial-to-mesenchymal transition (EMT) during which tumor cells acquire stem cell-like characteristics that enable invasion into the tissue. Microbes have been shown to directly activate signaling pathways involved in EMT (Arthur et al., 2012; Liu et al., 2023; Vincan and Barker, 2008; Rubinstein et al., 2013; Lu et al., 2014; Franco et al., 2005). In our study, melanoma cells pre-incubated with bacterial supernatant showed increased invasion and displayed a more mesenchymal morphology with increased dissemination to the tail fin. This phenotype was present despite washing off the bacterial supernatant from melanoma cells before injection, a result indicative of a transcriptional change. Downregulation of the transcription factor gene *Tfap2e* has previously been shown to mediate melanoma invasion and clustering (Campbell et al., 2021). However, we did not find consistent downregulation of *tfap2e* after incubation with bacterial supernatant (data not known) and, thus, other transcriptional regulators might be involved in this phenotype. We found that melanoma clustering was regulated by Rho GTPase signaling and, similarly, may drive invasive cell migration *in vivo*.

Our work suggests that lipids produced by *S. aureus* are responsible for changes in melanoma cell behavior. *In vitro* analysis determined that factors secreted by *S. aureus* directly affect the behavior of melanoma cell. Stripping off hydrophobic molecules with dextran-coated charcoal or Amberlite-XAD4 beads completely abolished any clustering effect of *S. aureus* supernatant. Furthermore, addition of bovine serum albumin increased cluster size 3-fold. Albumin proteins are known to bind serum lipids to increase cellular interactions with hydrophobic molecules in the blood (van der Vusse, 2009; Francis, 2010). Taken together, it is probable that lipids secreted by *S. aureus* promote melanoma clustering and invasion. However, we cannot exclude the possibility that other secreted factors, such as enzymes or metabolites play a role. Our work showed that only *S. aureus* strains USA300 and Newman promote melanoma clustering, suggesting that these strains comprise specific lipid-processing pathways that are not found in other Gram-positive or -negative species tested. Thus, we evaluated the role of specific *S. aureus* lipase mutants on melanoma clustering.

*S. aureus* express a selection of extracellular secreted lipases, including Sal2 that has previously been shown to alter immune cell behavior by inactivating pathogen derived ligands (Stoll et al., 2005). For example, Sal2 cleaves esterified fatty acids on bacterial lipoproteins to prevent TLR2-mediated immune recognition and subsequent cytokine production (Chen and Alonzo, 2019). In this study, mutation of *gehB* resulted in decreased melanoma clustering, suggesting that Sal2 lipase activity produces lipid mediators that are recognized by melanoma cells. Phosphatidylinositol (PtdIns)-specific phospholipase C (PIPLC; also known as PI-PLC) may work in a similar manner, as mutation of *plc* resulted in decreased melanoma clustering. PIPLC hydrolyzes membrane lipids such as PtdIns and membrane protein anchors containing glycosylphosphatidylinositol (Volwerk et al., 1990). Furthermore, PIPLC is highly expressed by *S. aureus* USA300 and Newman, but is not expressed by *S. epidermidis* (Volwerk et al., 1990), which correlates with our clustering data. Cleavage of these membrane lipids may produce mediators capable of interacting with and modulating melanoma cells. It is widely accepted that bacterial lipids can be recognized by adaptive T and NK cells during antigen binding on the CD1 receptor (Gras et al., 2018). Additionally, studies have indicated that they can be recognized by other eukaryotic and mammalian receptors, including GPCRs (Ryan et al., 2023; Cohen et al., 2017). In the gut, short-chain fatty acids (SCFAs) generated by bacterial fermentation can bind GPCRs to induce chemokine and cytokine production (Tan et al., 2014; Chun et al., 2019).

Future studies will be needed to apply these findings to human cancer cells. In addition, we did not evaluate the impact of live *S. aureus* bacteria. In previous work, *S. aureus* peptides have been found to bind HLA class I and II molecules within human melanoma cells (Kalaora et al., 2021), indicating that these bacteria may gain entrance and influence melanoma transcriptional activity intracellularly, similar to *F. nucleatum* (Casasanta et al., 2020).

Many questions still remain about whether bacterial skin colonization leads to disease. For example, *S. aureus* has been shown to activate mast cells and this heightened inflammation promotes the development of atopic dermatitis (Nakamura et al., 2013). However, more data support the theory that disruptions in the skin barrier leads to microbial dysbiosis (Yu et al., 2015; Zeeuwen et al., 2012; Grice and Segre, 2012). Skin barrier disruption results in chronic inflammation and has been correlated with development of non-melanoma skin cancers (Williams et al., 2020; Hoste et al., 2015). In cutaneous melanoma, ulceration is a negative prognostic factor and results in damage of the epidermal layer (Balch et al., 2009), which may allow for colonization of pathogenic species of *Staphylococcus*.

Here, we report that the skin microbe *S. aureus* promotes melanoma invasion and dissemination in a tumor transplantation larval zebrafish model. Our findings suggest that *S. aureus* lipase activity produces lipid mediators that modulate the behavior of melanoma cells, and supports further studies regarding the role of bacterial-derived lipids on cancer cell invasion.

## MATERIALS AND METHODS

### Ethics statement

Animal care and use protocol M005405-A02 was approved by the University of Wisconsin-Madison College of Agricultural and Life Sciences (CALS) Animal Care and Use Committee. This protocol adheres to the federal Health Research Extension Act and the Public Health Service Policy on the Humane Care and Use of Laboratory Animals, overseen by the National Institutes of Health (NIH) Office of Laboratory Animal Welfare (OLAW).

### Zebrafish lines and maintenance

Adult zebrafish and larvae (*Danio rerio*) were maintained as previously described (Vincent et al., 2016). Adult fish aged 3 months to 2 years were used to spawn larvae. Prior to experimental procedures, larvae were anesthetized in E3 water containing 0.2 mg/ml Tricaine (ethyl 3-aminobenzoate, Sigma). Larvae were maintained in E3 medium containing 0.2 mM N-phenylthiourea (PTU, Sigma Aldrich) from 1 day post fertilization (dpf) to prevent pigment formation during imaging experiments. All zebrafish lines used in this study are listed in Table S1.

### Bacterial strains and growth conditions

Bacterial strains used in this study are described in Table S2. Bacterial colonies of *S. aureus* were initiated on solid agar plates made with Tryptic soy agar (TSA; Sigma) or Luria broth (LB; Genesee) for other bacterial strains. Single colonies were picked and suspended in TSB or LB to initiate a liquid culture, followed by growing overnight at 37°C while shaking.

To generate bacterial supernatants, overnight cultures were diluted 1:100 into RPMI only medium and grown while shaking at 225 rpm until the optical density at 600 nm (OD_600_) was ∼0.9. For data analysis shown in Fig. 4A, we grew *S. aureus* in RPMI+2%FBS or RPMI+2%BSA. Cultures were centrifuged for 5 min at 3000***g*** and then filtered through a 0.2um SFCA filter to remove bacteria from the supernatant. Plated CFUs were used to normalize the filtered supernatant with the original culture medium to 3×10^8^ CFU/ml. Bacterial supernatant was aliquoted and frozen at −80°C for up to 3 months.

Mutant strains from the annotated Nebraska Transposon Mutant Library (NTML) generated in USA300 strain JE2 (BEI resources repository) were used in this study (see Table S2. For these strains, 2ug/ml erythromycin (Sigma) was used for antibiotic selection during overnight culture. Bacterial supernatants were generated in RPMI only medium. Prepared bacterial supernatants were utilized for zebrafish hindbrain injections or cluster formation assay as described below. The control USA300 JE2 strain was utilized for those experiments with transposon mutants (Fig. 5A-D).

#### Charcoal/Amberlite stripping

*S. aureus* supernatant was stripped off lipids and polar molecules by using dextran-coated charcoal or Amberlite XAD4. Dextran-coated charcoal (Sigma, catalog no. C6241) was prepared as previously described (Sikora et al., 2016). Briefly, charcoal was added at a final concentration of 0.25% (w/v) to distilled water (pH 7.4) with added 0.25 M sucrose, 1.5 mM MgCl2, and 10 mM HEPES and rotated overnight at 4°C. A volume of prepared dextran-coated charcoal was centrifuged at 500***g*** for 10 min to pellet the charcoal and the supernatant was aspirated. An equal volume of bacterial supernatant or RPMI only medium was added, vortexed to mix and incubated for 12 h at 4°C. The conical tube was centrifuged at 500***g*** for 10 min to retrieve the charcoal pellet.

Amberlite XAD4 (Thermo Fisher, catalog no. L14142.36) was also utilized to remove polar molecules from bacterial supernatant [previously described by Hansen et al., (2020)]. Amberlite beads (1% w/v) were measured out and washed during rotation in PBS for 3 h at room temperature. The beads were allowed to settle to the bottom and PBS medium was removed. Equal volume of bacterial supernatant or RPMI only medium was added and incubated overnight under rotation at 4°C. The prepared supernatant was pipetted into a new tube, filtered with a 0.2-μm filter and frozen at −80°C for up to 3 months. Supernatant stripped by using charcoal or Amberlite was added to ZMEL1 melanoma cells for cluster assay as described below.

#### Heat inactivation

*S. aureus* supernatant prepared as described above or *S. aureus* live bacteria were heated to 100°C for 15 min to denature/heat inactivate. Heat-inactivated bacteria were resuspended in RPMI only medium at 3×10^8^ CFU/ml. Heat inactivated supernatant or heat-inactivated bacteria were added to ZMEL1 melanoma cells for cluster assay as described below.

### ZMEL1 melanoma cell culture

We utilized ZMEL1-GFP or ZMEL1-tdTomato cell lines generated from a primary zebrafish melanoma model expressing BRAF V600E in a p53−/− background (Heilmann et al., 2015). Cell lines were tested for mycoplasma contamination. ZMEL1 melanoma cells were cultured in 10% FBS-DMEM supplemented with 1% Glutamax and 1% penicillin-streptomycin (ZMEL medium) on 10 µg/ml fibronectin coated plates in a sterile 28°C incubator. To harvest, cells were washed with sterile PBS, trypsinized and then counted with a hemocytometer.

### Injection of zebrafish ZMEL1 melanoma cells

Bacterial supernatants prepared in RPMI medium only were diluted 1:4 into ZMEL1 culture medium and incubated on the ZMEL1 melanoma cells for 3 days. Cells were harvested, washed in PBS, then resuspended in HBSS medium at a concentration of 8×10^7^ cells/ml. Cells were then loaded into thin-walled glass capillary injection needles. The needle was then calibrated to inject 1 nl (15–20 cells). Transgenic larvae were pre-screened for fluorescence using a zoomscope (EMS3/SyCoP3; Zeiss; Plan-NeoFluor Z objective). Anesthetized larvae were then placed on 3% agarose plates made with E3 and microinjected with ZMEL1 melanoma cells, with the time range set to milliseconds (ms) and pressure set to ∼15 PSI on the microinjector.

### Zebrafish imaging and quantification

#### Invasion assay

To assess invasion of ZMEL1 melanoma cells at the injection site, 2 dpi (days post injection) larvae were anesthetized and mounted in the zebrafish Wounding and Entrapment Device for Growth and Imaging (zWEDGI) device (Huemer et al., 2017) device such that the hindbrain was fully visible. Z-series of images (3.45-µm slices) of the hindbrain were acquired on a spinning disk confocal microscope (CSU-X; Yokogawa) with a confocal scan head on a Zeiss Observer Z.1 inverted microscope, Plan-Apochromat NA 0.8/20× objective, and a Photometrics Evolve EMCCD camera. Between imaging session larvae were kept in E3 with PTU in individual 24- well plates. Larvae that had tumor cells already separated from the injected cluster at 1 hour post injection (hpi) were excluded from the experiment.

#### Dissemination assay

At 4 dpi, larvae were scored as the percentage of larvae that exhibited tumor cell dissemination to regions posterior to the first somite, outside of the spinal cord, as previously described (Roh-Johnson et al., 2017). Larvae were screened for tumor cells in the trunk or tail at 3 hpi to eliminate fish to which ZMEL1 melanoma cells had been accidentally injected directly into the circulation. Screening and scoring of zebrafish larvae were done using a zoomscope (EMS3/SyCoP3; Zeiss; Plan-NeoFluor Z objective).

#### Neutrophil and Macrophage recruitment

Transgenic larvae were pre-screened for fluorescence using a zoomscope (EMS3/SyCoP3; Zeiss; Plan-NeoFluor Z objective). Larvae were anesthetized and mounted in the zWEDGI device (Huemer et al., 2017), such that the hindbrain was fully visible. Z-series of images (3.45-µm slices) of the hindbrain were acquired on a spinning disk confocal microscope. For neutrophil recruitment, images were taken at 1 hpi and 1 dpi. For macrophage recruitment, images were taken at 1 dpi and 2 dpi.

#### Image analysis and processing

Images of larvae represent a 3D rendering of the images generated on Imaris (v10.0). Invaded tumor cells were quantified as cells that were fully separated from the injected tumor cell cluster within the field of view. For neutrophil and macrophage recruitment, cells within 50 µm of the injected cluster were quantified.

### Cluster formation assay

Clusters were generated as previously described (Campbell et al., 2021). Briefly, ZMEL1-GFP melanoma cells were harvested and resuspended in 10% FBS-DMEM supplemented with 1% Glutamax and 1% penicillin-streptomycin. RPMI only medium (40 μl) or bacterial supernatant (40 μl) was first added to an ultra-low attachment 96-well plate (Corning, catalog no. 3474). Then, 5×10^4^ ZMEL1 melanoma cells per well were seeded in 120 μl on top to mix. Plates were incubated at 28°C for up to 7 days to allow clusters to form. Plates were imaged on indicated days using an inverted fluorescent microscope (Nikon Eclipse TE300) with a 20× objective and an automated stage (Ludl Electronic Products) with a Prime BSI Express camera (Teledyne Photometrics). Environmental controls were set to 28°C with 5% CO_2_. Fluorescent images were analyzed using ImageJ software. All data were quantified from culture day 7 onwards, unless otherwise indicated.

For movies of cluster formation, starting on day 5, ZMEL1 melanoma cell clusters were imaged every 30 min using the same imaging parameters described above. Videos were compiled using ImageJ software.

#### ROCK inhibition

To block cell migration during cluster formation, we used the selective Rho-associated protein kinase 1 and 2 (ROCK1 and ROCK2, respectively) inhibitor Y-27632 dihydrochloride (Bio-Techne, catalog no. 1254). RPMI only medium or *S. aureus* USA300 supernatant was supplemented with inhibitor to yield a final concentration of 500 nM. As described above, ZMEL1 melanoma cells were added on top to mix and incubated at 28°C for up to 7 days to allow clusters to form.

#### Pam3CSK4 and LPS

TLR agonists were added to melanoma cells for the cluster formation assay. Pam3CSK4 (Invivogen tlrl-pms) at final concentration 100 µg/ml or LPS from *E. coli* (Sigma, catalog no. L2755) at final concentration 1 µg/ml were added during setup of the cluster formation assay. As described above, ZMEL1 melanoma cells were added on top to mix and incubated at 28°C for up to 7 days to allow clusters to form.

#### Cluster quantification

The average cluster size of each image was quantified using ImageJ. Fluorescent EGFP images were processed as followed: *despeckled, auto thresholded*, converted to *binary* and the *area of particles* >30 µm were quantified. Any clusters on the edge of the image were excluded. A total of nine images were taken with three images per well and three wells per condition. At least 50 clusters were quantified per condition.

#### Viability staining and quantification

To measure viability of ZMEL1 cells in the cluster assay conditions, cells treated with medium alone or *S. aureus* supernatants were stained with propidium iodide (PI) (Biotium, catalog no. 40016) for 10 min. Cells were imaged on spinning disk (CSU-X; Yokogawa) with a confocal scan head on a Zeiss Observer Z.1 inverted microscope, Plan-Apochromat NA 0.8/10× objective, and a Photometrics Evolve EMCCD camera. A total of six images were taken with three images per well and two wells per condition. Images were processed using ImageJ software and segmented for ZMEL1 (EGFP) and PI using thresholding technique and background subtraction. Then, areas of ZMEL1 and PI were measured, and the average of each condition plotted.

#### Quantification of cluster motility

Motility analyses were performed on cluster movies from Day 5 using the *surfaces* function in Imaris (v10.0) to label the clusters. GFP signal was used to threshold and surfaces were generated with automatic tracking of motility over time. Further processing was performed to correct for drift. Clusters with track duration of <60 min and cluster size <200 (µm^2^) were filtered out. Average track mean speed of all the clusters from each movie were plotted from three movies.

### Statistics

All experiments with statistical analyses represent at least three independent replicates (*N*). Statistical significance was set to <0.05 and all statistical tests are two-tailed. The replicate number of zebrafish (*n*) for experiments in Figs 1C, D and 5D is indicated in the figure legend. Larvae from a single clutch were randomly assigned to the control or experimental group. Zebrafish were excluded from analysis if no ZMEL1 melanoma cells could be identified in the hindbrain following injection. For zebrafish experiments, equivariance was checked using an F test and determined that the samples did meet the requirement. We tested for outliers using the robust regression and outlier removal (ROUT) method (Motulsky and Brown, 2006), but no outliers were identified. Analysis of ZMEL1 melanoma cell invasion in zebrafish larvae was performed on data pooled from three independent experiments. *P-*values were calculated using unpaired *t*-test (Fig. 1C) or one-way ANOVA with Tukey's multiple comparisons (Fig. 5D). Analysis of ZMEL1 melanoma cell dissemination in zebrafish larvae (Fig. 1D) was performed on percent larvae with dissemination by using paired *t*-test.

Melanoma clustering with *S. aureus* supernatant versus heat inactivated bacteria, Gram-positive versus Gram-negative bacteria and the NE USA300 transposon mutants were analyzed by one-way ANOVA with Tukey's multiple comparisons. Melanoma clustering with *S. aureus* supernatant grown in the presence of FBS or BSA, or after treatment with ROCK inhibitor, dextran-coated charcoal or amberlite-XAD4, PI staining was analyzed by two-way ANOVA with Tukey's multiple comparisons Cluster motility movies were analyzed by unpaired *t*-test (GraphPad Prism version 10). All graphical representations of data were created in GraphPad Prism version 10 and figures were ultimately assembled using Adobe Illustrator (Adobe version 23.0.6).

## Supplementary Material

10.1242/dmm.050770_sup1 Supplementary information

Table S1. Zebrafish lines used in this study.

Table S2. List of bacterial strains used in this study.
