# Supplementary material for: Staphylococcus aureus lipid factors modulate melanoma cell clustering and invasion
Source: Dis Model Mech. 2024 Sep 16;17(9):dmm050770. doi: 10.1242/dmm.050770 (PMC11423913; doi:10.1242/dmm.050770)
Supplement: Supplementary information [file dmm-17-050770-s1.pdf]

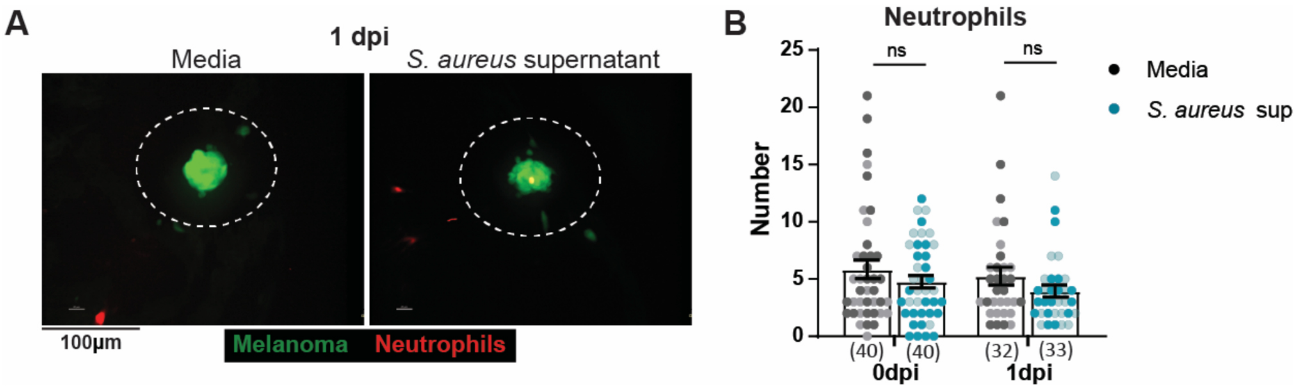

Fig. S1. Neutrophil recruitment to melanoma is not changed by incubation with *S. aureus* supernatant.

Neutrophil-labeled (*mpx:mCherry*) or macrophage-labeled (*mpeg1:gfp*) larvae were injected with EGFP or td-Tomato expressing ZMEL1 melanoma cells and imaged by confocal microscopy. (A) Representative images at 1dpi and (B) quantification of neutrophil recruitment to ZMEL1 melanoma cells. n=32-40 larvae per condition. Dots in (B) represent independent zebrafish larvae color-coded per replicate. Bars indicate the mean ± SEM. Dotted circles represent 50um region where recruited immune cells were counted. p values were calculated by Two-way ANOVA. ns – not significant.

Disease Models & Mechanisms • Supplementary information

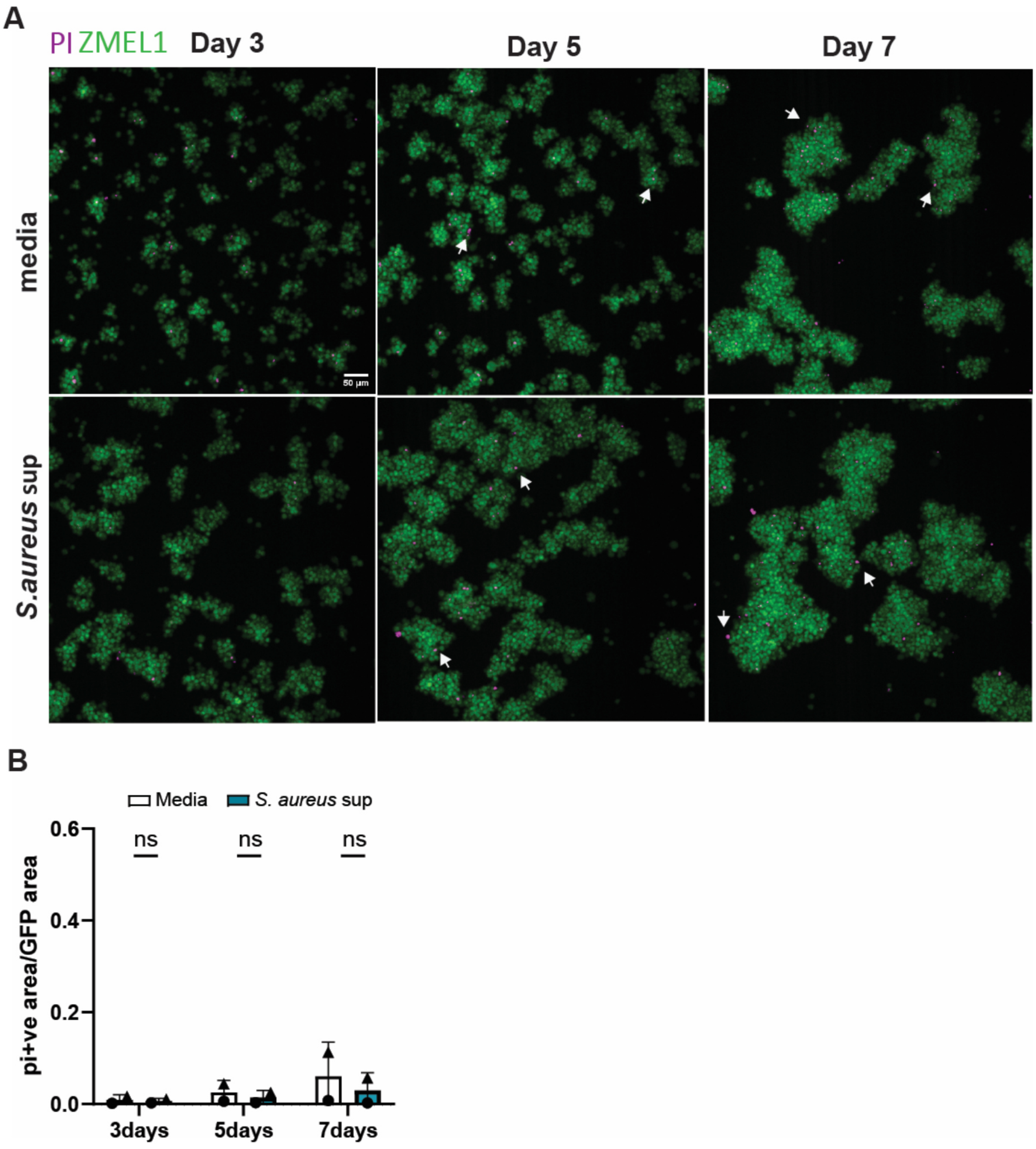

Fig. S2. Viability of melanoma cells is not altered with *S.aureus* sup treatment

(A) Representative images of ZMEL1-GFP cell clusters treated with either media alone or *S.aureus* supernatant at day 3, 5, 7 stained with propidium iodide for 10 minutes before imaging. (B) Quantification of fraction of pi+ve area to GFP+ area in the field of view. Dots indicate individual replicates and bars indicate mean± SEM. p-values were calculated by Two-way ANOVA. n.s – Not significant

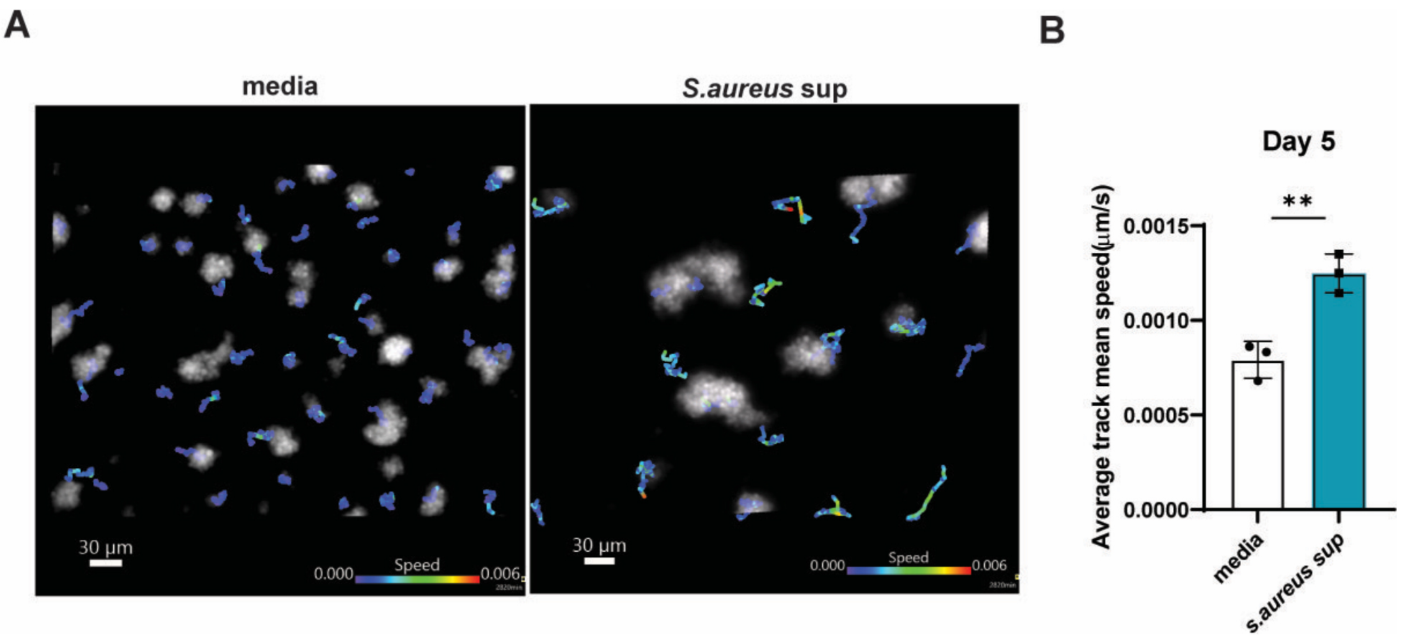

Fig. S3. (A) Representative images of cluster motility analyses for cells treated with media alone or S. aureus supernatant. Movies were taken on day 5 of culture every 30 minutes. Clusters were tracked and tracks are color coded for instantaneous speed. Cooler color indicates slower speed and warmer color indicates faster speed. (B) Quantification of average track mean speed of all 21 clusters in the field of view. 3 movies per condition were quantified and analyzed by unpaired t-test. ** - p<0.01.

Table S1. Zebrafish lines used in this study.

Available for download at
https://journals.biologists.com/dmm/article-lookup/doi/10.1242/dmm.050770#supplementary-data

Table S2. List of bacterial strains used in this study.

Available for download at
https://journals.biologists.com/dmm/article-lookup/doi/10.1242/dmm.050770#supplementary-data

References cited in Tables S1 and S2

Duthie, E. S. and Lorenz, L. L. (1952). Staphylococcal coagulase; mode of action and antigenicity. *J. Gen. Microbiol.* **6**, 95-107. doi:10.1099/00221287-6-1-2-95

Ellett, F., Pase, L., Hayman, J. W., Andrianopoulos, A. and Lieschke, G. J. (2011). mpeg1 promoter transgenes direct macrophage-lineage expression in zebrafish. *Blood* **117**, e49-e56. doi:10.1182/blood-2010-10-314120

Disease Models & Mechanisms • Supplementary information

Fey, P. D., Endres, J. L., Yajjala, V. K., Widhelm, T. J., Boissy, R. J., Bose, J. L., Bayles, K. W. (2013). A genetic resource for rapid and comprehensive phenotype screening of nonessential *Staphylococcus aureus* genes. *mBio* **4**, e00537-e00512. doi:10.1128/mBio.00537-12

Miller, L. G., Perdreau-Remington, F., Rieg, G., Mehdi, S., Perlroth, J., Bayer, A. S., Tang, A. W., Phung, T. O. and Spellberg, B. (2005). Necrotizing fasciitis caused by community-associated methicillin-resistant Staphylococcus aureus in Los Angeles. *N. Engl. J. Med.* **352**, 1445-1453. doi:10.1056/NEJMoa042683

Panagiotidis, C. A., Blackburn, S., Low, K. B. and Canellakis, E. S. (1987). Biosynthesis of polyamines in ornithine decarboxylase, arginine decarboxylase, and agmatine ureohydrolase deletion mutants of Escherichia coli strain K-12. *Proc. Natl. Acad. Sci. USA* **84**, 4423-4427. doi:10.1073/pnas.84.13.4423

Yoo, S. K., Deng, Q., Cavnar, P. J., Wu, Y. I., Hahn, K. M. and Huttenlocher, A. (2010). Differential regulation of protrusion and polarity by PI3K during neutrophil motility in live zebrafish. *Dev. Cell* **18**, 226-236. doi:10.1016/j.devcel.2009.11.015

Disease Models & Mechanisms • Supplementary information
